# Bringing Medical Care to the Streets: Lessons Learned in Launching a Street Medicine Outreach

**DOI:** 10.1002/aet2.70161

**Published:** 2026-04-15

**Authors:** Christine Shaw, Hanin Ali, Chase Palisch, Catherine Weaver, Erin F. Shufflebarger

**Affiliations:** ^1^ Department of Emergency Medicine The University of Alabama at Birmingham Birmingham Alabama USA; ^2^ Alix School of Medicine The Mayo Clinic Rochester Minnesota USA; ^3^ Vanderbilt University Nashville Tennessee USA; ^4^ University of California, Los Angeles Los Angeles California USA

**Keywords:** health services, homelessness, housing insecurity, social emergency medicine, street medicine

## Abstract

**Background:**

Street Medicine, where healthcare is provided in non‐traditional settings, provides care to people experiencing homelessness (PEH). Street medicine has been shown to improve patients' health and decrease Emergency Department visits, making it a cost effective way to provide medical care to PEH.

**Methods:**

This paper is based on the SAEM25 didactic session as conducted in Philadelphia, where a panel of providers who are directly involved in street medicine clinics shared their experiences and perspectives on the various designs in practice. These topics included key logistical challenges and solutions, barriers to care and trust building, strategies for fostering sustainable partnerships with community organizations, and options for medical student or resident involvement.

**Results:**

The design and unique approach of four different universities across the country was compared and contrasted by a panel of experts, all with experiences working directly with PEH. There are similarly identified barriers to providing care to PEH including location and transportation, financial and food insecurity, and trust between the community and the healthcare system.

**Conclusion:**

There are multiple options that exist to provide a framework to create a clinic to support populations of PEH.

## 1 Introduction

Persons who are living without fixed, regular, and adequate nighttime residence are described as homeless. Per the United States Department of Housing and Urban Development, an estimated 18 per ten‐thousand people experience homelessness on any given night [1]. People experiencing homelessness (PEH) experience significantly increased barriers to health care and subsequently have higher rates of chronic diseases [2, 3]. PEH experience high rates of violence, exposure to the environment, and robbery, leading to increased vulnerability [4]. Studies indicate that PEH experience mortality rates nearly 10 times higher than housed persons due to high rates of infectious diseases, cardiovascular disease, accidental injury, suicide, and substance use disorder [2, 3]. These barriers lead to an increased rate of hospitalization due to conditions which traditionally can be treated in an ambulatory setting (ambulatory care sensitive conditions [ACSCs]) such as asthma, diabetes, and urinary tract infections [5]. The Massachusetts Health Policy Commission published a Cost Trends Report in 2015 which estimated that at least 40% of Emergency Department (ED) visits during the study period were either non‐emergency or could have been managed in primary care [6].

Based on studies in California, about 9% of hospitalizations of PEH were due to ACSCs; these hospitalizations can possibly be prevented with increased availability of street medicine clinics [5]. Street medicine is a movement within medicine that provides healthcare to persons in non‐traditional settings such as in streets, parks, or under highway overpasses. It is designed to give targeted care to people living in extreme poverty, and especially for PEH [7, 8]. It is estimated that 60% of PEH do not have health insurance [2]. Street medicine clinics are novel in their approach and have been growing in popularity in the past decade, which fits the much‐needed population of PEH that has been growing throughout the United States [8]. Clinics such as these have been shown to improve the health of the participants in addition to defer ED visits, therefore making it a cost‐effective way to provide preventative medicine to PEH [9, 10, 11].

Social determinants of health (SDOH) are things outside of an individual's health that influence their health such as socioeconomic status, living conditions, and education level. According to the Centers for Disease Control (CDC) data suggests that health inequity leads to an extra 500,000 hospitalizations annually, costing an estimated $3.6 billion [6]. Mobile Health Clinics are shown to be impactful at providing care directly into vulnerable communities and can have an impact on the populations most disadvantaged by their SDOH [6]. Similarly, an analysis by Holmes et al. [12] demonstrated that access to and use of a dedicated homeless clinic was independently associated with decreased inappropriate ED visits. Street medicine clinics are a growing part of an evolving health care system, and can serve as a fiscally responsible stepping‐stone between the clinic and the community, addressing both medical and social need [2, 6].

Our SAEM25 didactic was intended to be an introduction to street medicine and to help identify opportunities to serve this vulnerable population. It allowed for persons directly involved with street medicine clinics to discuss their individual experiences, focusing on building trust with unhoused patients in order to help address the unique healthcare needs, key logistical challenges and solutions, the advantages/disadvantages of medical student and resident involvement, and strategies for fostering sustainable partnerships with community organizations.

## 2 Methods

This didactic was designed to discuss the variability of street medicine clinic structures and implementation based on the panel's expertise and experiences directly working at street medicine clinics which serve PEH. The presenting group, subsequently the authors of this summary, was assembled through the Society for Academic Emergency Medicine (SAEM)'s Social Emergency Medicine and Public Health (SEMPH) Interest Group, which also sponsored the didactic. Each author conducted individual literature reviews which supported the structure and implementation of their unique design for a street medicine clinic Table 1. These findings were then discussed collaboratively and integrated into a chart highlighting the different designs and the strengths and weaknesses of each design.

**TABLE 1:** Summary of program highlights.

	Mayo Clinic	UAB	UCLA	Vanderbilt
Location	Rochester, MN (population ~122,000)	Birmingham, AL (population ~200,000)	Los Angeles, CA. Divided into 8 Service Planning Areas (SPAs). Our teams serve SPAs 2, 4, 5, and 6, population of these SPAs: 4,885,294 (48,638 unhoused)	Nashville, TN (population ~710,000)
Structure	Established by the Zumbro Valley Medical Society in 2020 Initially an initiative to educate our community about homelessness through a series of online educational videos in 2020 Now includes a medical school longitudinal selective, multi‐specialty clinic, weekly street rounds, and a number of other related initiatives which now include people with lived expertise	Established by the *Equal Access Birmingham* (*EAB*) medical student‐run free clinic in March 2024 The clinic operates once monthly in a community park in partnership with *Be a Blessing Birmingham*, a nonprofit coordinating community organizations providing other services/support including clothing, food, substance use treatment, and peer support specialists The clinic utilizes a tent setup with portable chairs and tables, with medications and wound‐care supplies carried in a backpack. A mobile hotspot is used for laptop access to the EMR	Established as a health system based street medicine team formed in January 2022 Clinic is operated using 6 vans with teams staffed by health professionals including physicians, physicians assistants, nurse practitioners, registered nurses, medical assistants, social workers, case managers, and community health workers. Physicians come from a variety of specialties including emergency medicine, family medicine, internal medicine, and psychiatry	Established through the Vanderbilt Academic Medical Center, a hospital based homeless health service led by physicians and assisted by community health workers Patients recruited through ED screening for housing insecurity with connection to social workers Weekly street‐team rounds by multidisciplinary team
Student & residents	Students main avenue of participation is through the street medicine longitudinal selective in collaboration with Zumbro Valley Medical Society. Selectives are enrichment opportunities that are offered throughout the 4 years of medical school that can be utilized for career exploration, research, and any activity related to medical education. The street medicine selective is run by students in conjunction with a physician mentor and a partner from ZVMS. Students undergo and participate in 4 lectures/workshops. They then spend the rest of their hours volunteering through different opportunities including foot soaks, street runs, free eye clinics and free dermatology clinics	Student leadership: We are a student‐led clinic, as an extension of the student‐run free clinic. The student leadership team (in coordination with the faculty advisors) determines which medicines to carry and the scope of conditions to be treated, schedule student volunteers, and collaborate with community partners We incorporate medical students and residents as learners on the team under the supervision of a physician. Student volunteers conduct the intake, take vitals, perform the history and physical, create a treatment plan with the resident/attending physician, provide wound care, and document in the medical record	We incorporate medical students and residents as learners on the team under the supervision of a physician. Medical students are assigned as part of the Early Authentic Clinical Experience to rotate with our team. Resident physicians from emergency medicine rotate with us as part of a required social emergency medicine rotation during their PGY‐2 year. We also have family medicine residents who rotate as part of their community health rotation. We have served as a site for addiction medicine fellows	Currently, we offer non‐medical ways to for students to volunteer with our community partners. They participate in a foot washing clinic (and our street team evaluates for wounds). They coordinate vaccination efforts with health events that focus on our housing insecure, but coordinated by Metro government of local non‐profits. They also run a Street Medicine Interest Group SMIG, that provides education on a volunteer basis VUSM has not approved their participation outside of the hospital in any medical care. There has been approval for a student elective once the legal issues are resolved
Strengths	We serve a small population with the resources of a large medical center We are constantly working with and learning from those with lived experiences. For instance, the TABLE (Team of Advisors Bringing Lived Experience) is a team of individuals with direct experience of homelessness who provide input on programs, projects, and policies Medical students start gaining clinical exposure as early as their first year. The unique structure of selectives also makes it easy to incorporate new ideas, allowing the program to evolve each year based on student input. Recently, new additions have included a dermatology clinic and volunteer opportunities at a youth shelter	Partnership with medical student free clinic and SOM provides consistent funding for supplies and institution liability coverage This structure allows students to directly work with PEH and understand how various SDoH impact the way persons interact with their own health and the healthcare system There is an opportunity for medical learning, as a clinical teaching pearl is shared each session. Students follow patients from intake to discharge, therefore understanding the process within a clinic, receive hands‐on practice with taking vitals, completing a history and physical, and presenting a plan to an attending Patients receive direct care, meeting them where they are gathered in the park. With plans to expand services provided to be a more complete primary care clinic with POC testing	We have the support of a large health system Use of community health workers and enhanced case managers to improve outreach and collaboration with patients Behavioral health program with social workers and psychiatrists Enhanced care management program to support patient's care goals	We have the support of a major academic hospital system and associated services including HER/IT (Epic) support, social workers and case management Program utilizes a specific Program manager First CHWs involved which are expanding our capacity Strong partnerships and trust built with Community partners that are increasing our capacity Business model focusing on decreased capacity
Challenges	Funding and sustainability are ongoing challenges within our program, as we have largely relied on volunteers to deliver healthcare and related services to those in need Last summer, our city passed an ordinance which banned camping, and it has become increasingly challenging to find people who are experiencing homelessness and meet them in their own spaces. Winter in Minnesota can be a challenge for everyone, especially our unhoused population. We continue to explore and seek options so that everyone has a safe place to live	Limited scope of practice at this time, which feels restrictive in what we can treat—medications distributed are selective of just those in our bag, therefore a very limited formulary is available, and sometimes we run out of certain medications The community is not completely in support of our clinic operating in the park, as they feel that it is “attracting homeless” to be in that area and make it unsafe. Due to this police are sometimes called, and the park was “cleaned out” and all homeless displaced this fall	Integrating medical and social models to provide holistic care to our patient population Funding and sustainability Navigating LA County's complicated Medicaid system Oppressive sweeping practices Communication and collaboration amongst many Los Angeles street medicine teams	More work than capacity—significant need in the community Our system is hospital based. We have the responsibility to demonstrate financial sustainability and value to the hospital system. Thus far we have been able to leverage humanitarian needs, equity goals and service learning opportunities. We have also aligned goals to decrease hospital capacity. We believe our service can decrease the financial burden of the hospital as we decrease the number of ED visits, complications from lack of follow up and decrease LOS once admitted We also faced the challenge of proving our street efforts would be safe to a conservative legal team prior to launch. It was a long process, but wins were gained as we leveraged current practices and compared to parallel processes at other institutions that had been approved and working successfully

The presenting panel was composed of the authors of this article, whose backgrounds span the academic spectrum from academic research, education, and clinical experience including a medical student: Dr. Erin Shufflebarger and Dr. Christine Shaw are the co‐medical directors of the University of Alabama (UAB) Street Medicine Clinic, Dr. Catherine Weaver is the medical director of the University of California Los Angeles (UCLA) Health Homeless Healthcare Collaborative, Dr. Chase Palisch is the Assistant Director of Vanderbilt University Medical Center (VUMC) Homeless Health Services, and 4th year medical student Hanin Ali is the Co‐Leader of Street Medicine Selective/Education at the Mayo Clinic.

The conceptual framework of the panel was focused on the unique locations of the clinics, spanning four different states, the various structures and implementation of each clinic (walking, mobile bus, etc.), the varying involvement of learners, and the unique challenges faced based on the structure and implementation technique. Our shared knowledge and experience shaped the distinct insights and educational suggestions provided in this work.

## 3 Discussion of Didactic Panel

The four academic health centers represented in this panel have four different approaches to street medicine, as described below. The panel discussed the various differences within each environment, clinic structure, and strengths of the design:

### 3.1 UCLA

The UCLA Health Homeless Healthcare Collaborative (HHC) uses 6 mobile vans to provide urgent care, transitional primary care, and behavioral health services within 4 of the 8 Service Planning Areas of Los Angeles (LA) County [13]. LA County is an expansive 4060.3 mile^2^ with a population of 10,014,009 per 2020 census data [14]. Per the 2025 Point‐in‐Time Count, there are 72,308 PEH in Los Angeles County, 47,413 of these PEH are unsheltered [15]. HHC was established in January 2022 with the goal to promote greater health equity and improved clinical outcomes for PEH in Los Angeles County by meeting patients where they are and partnering with them and the community to achieve health and social justice for those experiencing homelessness [13]. The UCLA clinic uses an interdisciplinary team including physicians, physician assistants, nurse practitioners, registered nurses, medical assistants, social workers, case managers, and community health workers, which operates daily. In alignment with their commitment to equity, the team members are paid per fair market value. This clinic serves patients experiencing both sheltered and unsheltered houselessness directly on the streets, in encampments and shelters, and at access events, providing urgent care and transitional primary medical care, behavioral health services, and assistance with social determinants of health. Mobile units are stocked with pharmaceuticals for dispensation, lab equipment, wound care equipment, point‐of‐care ultrasounds, and outreach supplies. The team documents in Epic, the Electronic Medical Record system used by the health system [13].

Medical students and residents have opportunities as learners on the team under the supervision of a physician or advanced practice provider. First‐year medical students are assigned as part of the longitudinal Early Authentic Clinical Experience curriculum to rotate with the team. Resident physicians from emergency medicine rotate as part of a required social emergency medicine rotation during their PGY‐2 year. In addition, family medicine residents rotate as part of their community health rotation. Additional options exist for addiction medicine fellows and for psychiatry interns [13].

This clinic is strengthened by the large network of support from UCLA Health hospitals and infrastructure as well as by partnerships with community organizations and mutual aid groups that provide referrals and assist with social support. The interdisciplinary approach includes community health workers and enhanced case managers to improve outreach and collaboration with patients and a Behavioral health program with clinical social workers and psychiatrists. Medical and social models are integrated to provide holistic, person‐centered care to our patients [13].

### 3.2 UAB

The UAB street medicine clinic was established in March 2024, as an extension of the Equal Access Birmingham (EAB), a student‐led free clinic [16]. Equal Access Birmingham has existed for over 10 years with the mission to provide care to the uninsured and underserved populations in the greater Birmingham area while also offering a meaningful service‐learning experience to augment the medical education at the UABHSOM. EAB already has established grant and fundraising support, a medication distribution license, and a liability structure, which was easily expanded to cover the street medicine initiative. The street medicine clinic operates once monthly in a community park in partnership with Be a Blessing Birmingham, a nonprofit coordinating community organizations providing other services/support including clothing, food, substance use treatment, and peer support specialists. The clinic utilizes a tent setup with portable chairs and tables, with medications and wound‐care supplies carried in a backpack. Due to these limitations, the clinic primarily offers urgent/convenience care needs such as wound care, cellulitis, and abscesses. The population served is primarily those who live in encampments in/near the park [16].

As EAB is a student‐led clinic, there is a student leadership team who, in coordination with the faculty advisors, determines which medicines to carry and the scope of conditions to be treated, schedules student volunteers, and collaborates with community partners. Student volunteers conduct the intake, take vitals, perform the history and physical, create a treatment plan with the resident/attending physician, provide wound care, and document in the medical record. This structure allows students to directly work with PEH and understand how various SDoH impact the way persons interact with their own health and the healthcare system. In addition this allows the student to follow a patient from intake to discharge, therefore understanding the process within a clinic; receive hands‐on practice with taking vitals, completing a history and physical, and presenting a treatment plan to an attending [16].

### 3.3 VUMC

The VUMC is a hospital based homeless health service in Davidson County, Tennessee, led by physicians and assisted by community health workers [17]. Patients who present to the ED are screened for homelessness and housing insecurity, and are then connected to the social work team and street outreach team. The team is able to perform weekly rounds, traveling to several homeless encampments throughout Nashville, for those unable or unwilling to access a traditional clinic in the community or VUMC. This program benefits from the hospital level support, space, and organizational power that comes from partnering with a large university hospital [17].

Currently, students are able to volunteer in non‐medical ways with community partners, such as the foot‐washing clinic at Room In the Inn, help coordinate vaccination efforts with health events that focus on our housing insecure in conjunction with the Metro government of local non‐profits, and have educational sessions through a Street Medicine Interest Group (SMIG) at the medical school [17].

### 3.4 Mayo Clinic

The Zumbro Valley Medical Society (VZMS) established a street medicine clinic in 2020 to serve the Rochester, Minnesota population [18]. What began as an educational series about homelessness for the community, became a longitudinal curriculum in the medical school, then led to special efforts in multi‐specialty clinics, street rounds, and a number of other related initiatives which now include people with lived expertise. One of the biggest and most unique advantages of this clinic is the partnership with people with direct experience of homelessness who provide input on programs, projects, and policies (TABLE [Team of Advisors Bringing Lived Experience]) [18].

Students are able to complete a longitudinal selective in collaboration with ZVMS. Selectives are enrichment opportunities offered throughout the 4 years of medical school that can be utilized for career exploration, research, and any activity related to medical education. The street medicine selective is run by students in conjunction with a physician mentor and a partner from ZVMS. Students participate in 4 lectures/workshops, and then spend the rest of their hours volunteering through different opportunities including foot soaks, street runs, free eye clinics, and free dermatology clinics [18].

The Mayo street medicine program operates on a small scale and is largely dependent on the schedule of the emergency medicine physician who leads the initiative. Outreach typically occurs once per month, with greater frequency during the summer and fall when the population is more accessible. The outreach team generally includes the physician, a medical student, and a social worker who travel using the physician's personal vehicle. There is no fixed clinic space; care is provided outdoors at encampments or in collaboration with local organizations such as The Landing, Rochester Warming Center, Salvation Army, and Dorothy Day. These partnerships are informal and not structured around scheduled clinic days. Medical resources are sourced from donations and are limited to supplies for basic acute care. When additional needs arise, the team identifies external community resources or arranges to provide necessary items at a later time. Medical students assist with patient assessments under supervision but do not perform independent procedures or formal case presentations. The primary objectives of each visit are to build rapport with individuals experiencing homelessness, address immediate health concerns within the constraints of available resources, and connect patients to appropriate long‐term health and social services [18].

### 3.5 Limitations

While each group has a unique design and location specific demographics, there are several common challenges with working with PEH. There are similar barriers experienced by the population such as the lack of sanitation, food security, and personal security; PEH report high rates of victimization and theft [3]. PEH have a more difficult time storing their medications and are limited to those which don't require temperature control. Furthermore, PEH have similar barriers to care such as cost and transportation limitations [2, 3]. Maintaining legal documentation such as a driver's license, and health literacy to complete Medicaid or office intake forms further limit access [4].

These panelists have all acknowledged difficulties with the limitations of providing care in the streets such as the limited scope due to decreased access to advanced testing, limited medication supplies, and the overwhelming need that far exceeds the resources available. Moving forward, securing sustainable funding to ensure this vulnerable population has access to care is an obstacle.

Each location will also face challenges that are specific to their region such as state and city legislation. Some laws even outlaw sleeping in public spaces or setting up tents, which allow for police to remove PEH from their spaces, leading to loss of belongings and criminal records. Oppressive sweeping practices can significantly derail progress that patients have made in their medical and social goals due to loss of communication methods, medications, legal documents, and more [4]. Programs designed to move people into interim housing may provide housing elsewhere in the county far from where they had been living, which can disconnect patients from their medical and social support networks [4, 19].

In Birmingham, where the population is primarily people with SUD, there is a strong mistrust of the hospital system due to prior negative interactions and failure to prevent/treat withdrawals while hospitalized. Additionally, the community is not completely in support of the clinic operating in the park, as they feel that the clinic is “attracting homeless” to be in that area and make the area unsafe. As a result, the police are sometimes called, and the park “cleaned out” displacing all the PEH who stayed there; some found permanent housing, some have come back, and some have found new camping sites.

In Nashville, there is a rapidly changing population. As the city grows and expands, there are further efforts to break up encampments, and therefore patients become physically lost. Nashville specifically has made sleeping on public property a felony [20]. The structure of the clinic, being connected to an academic institute, adds the pressure of billing and the necessity to demonstrate financial sustainability, thus adding value to the hospital system. Thus far the clinic has been able to leverage humanitarian needs, equity goals, and service learning opportunities, with the goal to decrease the financial burden of unnecessary ED visits, complications from lack of follow up, and decreased level of service once admitted [17].

In Minnesota, this clinic faces the unique challenges of severe winter weather compared to the other locations. In 2024 the city passed an ordinance which banned camping, and it has become increasingly challenging to find people who are experiencing homelessness and meet them in their own spaces [21].

In California, the significant size and the number of PEH and communication and collaboration amongst many Los Angeles street medicine teams has proven to be a challenge. There are significant limitations to health information exchanges, and it can be difficult to determine when other teams are caring for patients [19]. The mobile clinic discussed is multidisciplinary, which is both a challenge and strength, as prioritizing social versus medical goals in a way to maximize skill sets and utilize resources is a balancing act [13]. LA County has a complicated Medicaid system that includes a Fee‐for‐Service plan and different managed care plans (MCPs) that subcontract with different Independent Practice Associations (IPAs) to create their provider network [15]. These groups similarly have variable contracts with different hospital systems, which can make providing support for our patients complicated.

## 4 Conclusion

Street medicine has the potential to make a significant impact on PEH, even in locations with oppressive legislation. This population is especially vulnerable and has difficulties navigating the current healthcare system. There are multiple models that are effective to reach this population including mobile bus clinics, walking clinics, and medical‐student clinics (Appendix S1). Social determinants of health play a major role in healthcare outcomes, and hands‐on experience with this population can make a significant impact on an early learner.

## Author Contributions

All authors contributed to the conceptualization and design of this didactic. All authors contributed to the drafting and revision of the manuscript. All authors are responsible for the content of and have approved the manuscript as submitted.

## Funding

There was no funding directly related to this presentation at SAEM or creation of this manuscript.

## Conflicts of Interest

The authors declare no conflicts of interest.

## Supporting information


**Appendix S1:** Supplemental information for establishing a clinic for PEH.

## Data Availability

Data sharing not applicable to this article as no datasets were generated or analysed during the current study.
