# Supplementary material for: Bringing Medical Care to the Streets: Lessons Learned in Launching a Street Medicine Outreach
Source: AEM Educ Train. 2026 Apr 15;10(2):e70161. doi: 10.1002/aet2.70161 (PMC13083216; doi:10.1002/aet2.70161)
Supplement: Supplementary file 1 — Appendix S1: Supplemental information for establishing a clinic for PEH. [file AET2-10-e70161-s001.docx]

## **Appendix1. Supplemental information for establishing a clinic for PEH**

## **Helpful Resources**

- Society of Student-Run Free Clinics (SSRFC): <https://www.studentrunfreeclinics.org/our-story/>
- Street Medicine Institute: <https://www.streetmedicine.org/>
- National Association of Free & Charitable Clinics (NAFCC): <https://nafcclinics.org/get-involved/start-a-clinic/?utm_source=google&utm_medium=paidads&utm_campaign=607609838&utm_term=&utm_content=63778165168&gad_source=1&gclid=Cj0KCQiA4-y8BhC3ARIsAHmjC_Ga1ZGvDkEYfKvTUMKO3yXqDFz7O27SmJVQ36whUKyVdD3h7Dv_uHEaAoUlEALw_wcB>
- Steps to starting a free clinic: <https://familymedicine.ucsd.edu/education-training/freeclinic/education/start-free-clinic/how-to.html>

## **Things to consider**

- Medical - legal liability, malpractice coverage
- Safety considerations - need officer presence, group setting, emergency response plan
- Scope of practice - limitations of poc testing, lack of follow up, inconsistent presence of patients
- Funding for supplies - consistency
- Medications - scripts vs medication distribution (Distribution license)
- Lab testing - CLIA-waived tests, partnership with testing organization (partnering university, or Labcorp at cost)
  - <https://www.cdc.gov/clia/docs/tests-granted-waived-status-under-clia.pdf>
- Referral pathways for advanced studies, specialty care, hospitalization if necessary
- Importance of community health workers and utilizing team members with lived experience, but also setting up a supportive work environment for them
- Understanding the landscapes of services already offered in your community and how to collaborate and enhance/expand these efforts but not duplicate
